# Recombinant Human Enterovirus 71

**DOI:** 10.3201/eid1008.040059

**Published:** 2004-08

**Keywords:** Enterovirus 71, Coxsackievirus A16, hand, foot and mouth disease, recombination, dispatch

## Abstract

Two human enterovirus 71 (HEV71) isolates were identified from hand, foot and mouth disease patients with genome sequences that had high similarity to HEV71 (>93%) at 5´ UTR, P1, and P2 and coxsackievirus A16 (CV-A16, >85%) at P3 and 3´UTR. Intertypic recombination is likely to have occurred between HEV71 and CV-A16 or an as-yet to be described CV-A16-like virus.

Hand, foot and mouth disease (HFMD) is a common illness of infants and young children <10 years of age. It is characterized by fever, ulcers in the oral cavity, and rashes with blisters that appear on the palm and sole. The most common causal agents of HFMD are coxsackievirus A16 (CV-A16) and human enterovirus 71 (HEV71), but other enteroviruses, including CV-A5 and CV-A10, can also cause it. When caused by CV-A16 infection, it is usually a mild disease, and patients normally recover without requiring any special medical attention.

In rare instances, the disease leads to aseptic meningitis and more serious diseases, such as encephalitis or poliomyelitis-like paralysis. HFMD caused by HEV71 has been associated with the more severe forms of the disease, including a high number of cases of fatal encephalitis during the outbreaks in Malaysia in 1997 and Taiwan in 1998. During these outbreaks, several HEV71 subgenotypes were isolated; two subgenotypes, B4 and C2, were identified as the main causal agents associated with the fatal infections (1–3). During the Malaysia 1997 outbreak, HEV71 subgenotype B3 was the most prevalent subgenotype isolated from patients with the milder form of HFMD (4). The B3 virus was also found in neighboring countries in 1997 and western Australia in 1999; it has since disappeared (4–6).

To elucidate the mechanisms underlying the emergence of the different HEV71 subgenotypes with potentially different pathogenic potentials, we examined the whole genome sequence of representative HEV71 isolates of HFMD patients from the Malaysia 1997 outbreak. Virus was isolated and identified from patients' samples as described earlier (1). Initial characterization and construction of phylogenetic trees was performed by using the virus genome 5´ nontranslating region (NTR) sequences. From this initial tree, six isolates (UH1 [GenBank accession no. AJ238455], SHA89 [AJ586873], SHA63 [AJ238456], SHA66 [AJ238457], SHA52 [AJ238531], and SHA71 [AJ238535]) were randomly selected to represent the different HEV71 genotypes and were sequenced in their entirety. Sequence analysis, construction of phylogenetic trees, and potential recombination of the isolates were determined as previously described (1,7). Similarity plot and bootscan analyses for the recombination studies were performed with SimPlot version 3.2 (8,9). For the analysis, a sliding window of 400 nt was moved in increments of 20 nt at a time. Sequences were not corrected for multiple substitutions, all gaps were stripped, transition-to-transversion ratio of two was used, and 50% consensus files were used to exclude the poorly conserved sites. Recombination was identified when conflicting genome sequence profiles appeared, which suggested acquisition of sequences from a different parental genotype. Phylogenetic trees were then constructed for each of the putative recombinant sequences by using the maximum likelihood method, and support for the tree topology was determined by bootscanning analyses that used the bootstrapping procedures (9) with 100 resamplings. The crossover breakpoints were identified when χ^2^ values were maximum (10). In addition, recombinant sequences were confirmed in patients' samples by using the reverse transcription–polymerase chain reaction (RT-PCR) with primers RECF (5´-CTCAACAGAGCTGTGCTAGTCATGCAATCC-3´; nucleotide positions 5229–5258) and RECR (5´-TCCACTGAGGTTGAGAAAACCATATTGCAC-3´; nucleotide positions 5748–5777), designed on the basis of isolates SHA63 and SHA66 genome sequences and DNA sequencing.

Initially, a phylogenetic tree depicting the genetic relationships of the isolates was constructed by using the whole genome sequence of the six isolates and those available in the GenBank (Figure 1A). As expected, the six isolates were placed into three different lineages (genotypes), and as no reports exist on the typing of the different HEV71 isolates with the whole genome sequences, the genotypes established with the virus capsid (VP1) gene sequence were adopted (5). The three HEV71 subgenotypes identified, B3, B4, and C2, represent the HEV71 genotypes found cocirculating in Malaysia during the 1997 outbreak. Sequence analyses performed by using the isolates whole genome sequences initially reaffirmed the genotyping of the isolates (Figure 1A). The isolates remained within the respective genotypes when the P1 and P2 genome regions were used to construct the phylogenetic trees (Figure A1). A significant shift in the tree topology, which involved the positions of the two subgenotype B3 isolates, SHA63 and SHA66, was noted when the tree was constructed by using the P3 genome region, a region consisting of the nonstructural protein genes located towards the 3´ end of the genome (Figure 1B). At this genome region, these isolates clustered with the other known causal agent of HFMD, CV-A16. This conflicting tree topology raised the possibility that these isolates contained chimeric genome sequences, perhaps as a result of a previous recombination event involving HEV71 and a human enterovirus A (HEV-A). We performed similarity plot analysis using the consensus genome sequence of HEV71 subgenotype B3 isolates against several potential parental genomes, including all available HEV-A, and confirmed conflicting genome profiles resembling a pattern of recombination for the two subgenotype B3 isolates (Figure 2). The isolates showed high sequence similarity (>93%) to subgenotype B4 at the 5´ terminus of the genome spanning the 5´ NTR region, the whole structural gene sequences, 2A and part of 2B gene (nt 1–3908). A conflict in the genome sequence showing high similarity (>85%) to CV-A16/G10 was noted at the 3´ end of the genome involving the 3C protease, 3D polymerase, and 3´ NTR region gene sequences. Two estimated crossover points that resulted in a switch from HEV71 genotype B to CV-A16–like sequences (p < 0.001, Fisher exact test) were located within nt 3908–5603. However, the B3 genome sequence dissimilarity relative to subgenotype B2 and B4 viruses began as early as at nt 3617 (part of 2A gene), which does not rule out the possibility that this may be the first crossover point, even though the χ^2^ values supporting the crossover point was most statistically significant at position 3908.

**Figure 1:**
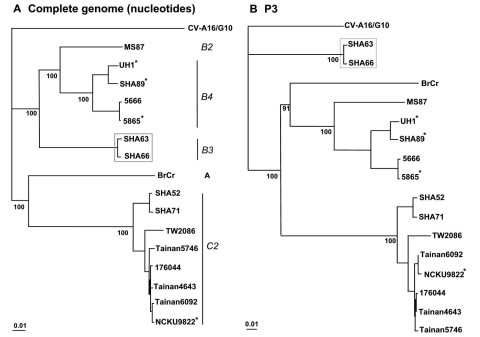
Phylogenetic trees showing genetic relationships among Human Enterovirus 71 (HEV71) isolates. The neighbor-joining trees were constructed from alignment of the whole genome sequences (panel A) and nucleotide sequences of P3 (nucleotides 5067­7325) (panel B). The bootstrap values are shown as percentage derived from 1,000 samplings, and the scale reflects the number of nucleotide substitutions per site along the branches. Isolates from fatal cases are denoted with asterisks.

**Figure 2:**
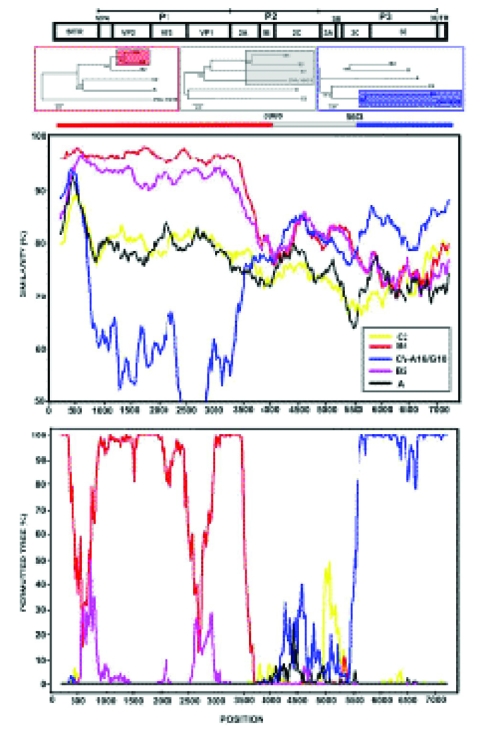
Identification of recombinant sequences in the genome of Human Enterovirus 71 (HEV71) subgenotype B3. A) shows the conflicting tree topology at the P3 genome region. B) shows results from similarity plot and bootscan analyses indicating the recombination sites. The window size of 400-nt slides in increments of 20 nt at a time. Positions containing gaps were excluded from the comparison.

Locating a definite breakpoint was not possible within the B3 virus genome region, as the genome region consisting of part of 2B, 2C, 3A, 3B, and part of 3C genes had a more complex genome sequence with weak association (low bootstrap values) to all other HEV71 and CV-A16 isolate sequences. Hence, the genome region served as a distinct signature sequence for the subgenotype B3 isolates.

Specific RT-PCR for detecting CV-A16 VP1 (CV-A16-VP1), HEV71 VP1 (HEV71-VP1), and HEV71/CV-A16-like sequence (REC) performed on the patients' specimens amplified only enterovirus sequences from all the samples by using the generic enterovirus primer sets that amplified the 5´ NTR (11). This finding suggests that all the samples, except for the controls, contained enterovirus sequences. In contrast, only HEV71-VP1 and HEV71/CV–A16-like sequences were amplified from SHA63 and SHA66 samples by using the respective PCR amplification primers (data not shown). No CV-A16-VP1 sequence was detected in either SHA63 or SHA66 patients' samples. These results suggest that the CV-A16–like sequences were not likely to have arisen from a serendipitous sequence amplification artifact involving samples with dual infections with HEV-71 and CV-A16. Furthermore, as both of the B3 isolates were obtained from two different HFMD patients (1), the existence of subgenotype B3 HEV71 with CV-A16–like sequences in nature is highly supported.

Further examination of all available 3D polymerase gene sequences (390 nt, subgenotype B3 nucleotide position 6696–7085) of all human enterovirus A (HEV-A) associated with HFMD (12), CV-A5, CV-A10, CV-A16, and HEV71, showed that subgenotype B3 isolates SHA63 and SHA66 had consistently higher nucleotide sequences similarity to CVA-16 (86%) than to all other HEV71 (75%–81%) and HEV-A (76%–78%) (Table A1). The presence of this CV-A16–like sequence within the B3 virus genome suggested that a possible recombination event had previously occurred between HEV71 and CV-A16. The finding that the two viruses tend to cocirculate within the same population during most HFMD outbreaks (13) supported the likelihood that this recombination could have happened. Nonetheless, the parent virus may be a yet-to-be described HEV-A with high sequence similarity to CV-A16.

The CV-A16-like protease and polymerase gene within the B3 virus isolates genomes could influence the pathogenic potentials of the virus in humans. The B3 virus was likely less pathogenic as the B3 isolates, SHA63, and SHA66, were obtained from uncomplicated HFMD cases, and the B3 virus was not the main virus isolated from children with severe diseases during the outbreaks in Sarawak, Singapore, Malaysia, and Perth (4–6). However, whether all other B3 viruses shared similar characteristics is not known.

Recombination among nonsegmented RNA viruses was once thought to be uncommon. However, findings involving HIV, dengue virus, and poliovirus have established that intratypic recombination does occur among nonsegmented RNA viruses. Similar to enteroviruses (14) and dengue viruses (7), recombination could result in the emergence of viruses with altered pathogenic potentials. In our study, the discovery that two B3 isolates could have emerged as a result of a previous recombination event raises the possibility that recombination events among enteroviruses associated with HFMD occur more frequently in nature. These recombination events could be the mechanism driving the emergence of a number of newly described HEV71 lineages in Asia, some with differing pathogenic potentials.

This study is funded in parts by grants from the Ministry of Science, Technology and Innovation, Malaysia # 06-02-09-001-BTK/TD/002.

## Figures and Tables

**Figure A1:**
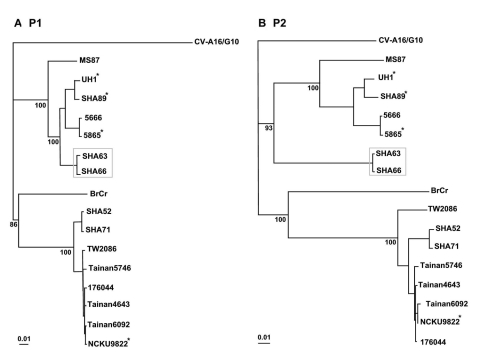
Phylogenetic trees showing genetic relationships among Human Enterovirus 71 (HEV71) isolates. The neighbor-joining trees were constructed from alignment of nucleotide sequences of P1 (nt 747–3332) (panel A) and P2 (nt 3333–5066) (panel B). Bootstrap values are shown as percentage derived from 1,000 samplings, and the scale reflects the number of nucleotide substitutions per site along the branches. Isolates from fatal cases are denoted with asterisks.

## Appendix

**Table A1:** Comparative nucleotide sequence analysis of partial 3D polymerase

HEV71 Isolates	CV-A16/G10	CV-A14	Sha63	Sha66	CV-A5	CV-A2	CV-A10	CV-A12	CV-A7	BrCr	MS87	UH1	Sha89	5666	5865	Sha52	Sha71	TW 2086	NCKU 9822	Tainan4643	Tainan5746	Tainan6092	176044
CV-A16/G10		86	86	86	78	75	76	75	76	76	76	80	80	80	80	81	81	80	80	80	80	80	80
CV-A14			83	83	79	77	76	76	75	78	76	77	77	77	77	80	80	80	80	80	80	80	80
Sha63				100	78	76	76	78	76	75	77	78	77	78	79	81	80	80	80	80	80	80	81
Sha66					78	76	76	78	76	75	77	78	77	78	79	81	81	80	81	81	81	81	81
CV-A5						83	82	80	81	79	84	84	83	84	84	84	83	83	84	83	83	83	83
CV-A2							90	86	81	85	82	82	82	83	83	81	81	81	81	82	82	82	82
CV-A10								86	81	83	81	82	82	82	82	79	79	80	80	80	80	80	80
CV-A12									82	86	82	81	80	83	83	80	81	81	81	80	80	80	80
CV-A7										81	81	82	82	83	83	82	82	81	80	81	81	81	81
BrCr											79	80	80	81	81	80	80	80	80	80	80	80	80
MS87												87	87	88	89	80	81	79	79	79	79	79	79
UH1													98	96	96	82	81	81	81	81	81	81	81
Sha89														95	95	82	81	80	81	81	81	81	81
5666															99	81	81	80	80	80	80	80	80
5865																81	82	81	81	80	80	80	80
Sha52																	99	95	95	96	96	96	96
Sha71																		96	95	96	96	96	96
TW2086																			99	99	99	99	99
NCKU9822																				99	99	99	99
Tainan4643																					100	100	100
Tainan5746																						100	100
Tainan6092																							100
176044																							
